# Irrigation Solutions in Wound Care and Breast Surgery: Evidence-Based Applications, Regulatory Considerations, and Future Directions

**DOI:** 10.3390/jcm14217679

**Published:** 2025-10-29

**Authors:** Stephanie M. Mueller, LaYow C. Yu, Michael Drake Pike, Hannah D. Shi, Dennis P. Orgill

**Affiliations:** 1 Division of Plastic and Reconstructive Surgery, Brigham and Women’s Hospital, 75 Francis St, Boston, MA 02115, USA; smmueller@mgh.harvard.edu; 2 Harvard Medical School, 25 Shattuck St, Boston, MA 02115, USA; layow_yu@hms.harvard.edu (L.C.Y.); drake_pike@hms.harvard.edu (M.D.P.); hannah_shi@hms.harvard.edu (H.D.S.)

**Keywords:** wound irrigation, saline irrigation, antiseptic irrigation, surgical site infection/prevention and control, breast pocket irrigation, Povidone–Iodine (PVI), Polyhexamethylene Biguanide/polyhexanide (PHMB), Hypochlorous Acid (HOCl), Sodium Hypochlorite (NaOCl), Triple antibiotic solution (TAS)

## Abstract

Background: Irrigation is a key strategy in reducing bioburden, disrupting biofilms, and supporting wound healing. While saline is the standard for its safety and availability, antiseptic and antibiotic solutions are often used in clinical scenarios that require infection control. However, the rise in antibiotic stewardship and concerns regarding cytotoxicity are reshaping current practices. This review identifies recent trends, current controversies, and persistent gaps in knowledge that warrant further investigation and regulatory attention. Methods: A literature review identified irrigation solutions commonly used in plastic surgery; labeling and concentrations were obtained from Devices@FDA, Drugs@FDA, and DailyMed, and PubMed, Cochrane Central, and Embase were searched (January 2022–July 2025) for human studies on acute wounds, chronic wounds, and implant-based breast surgery. Results: In acute wounds, saline and potable tap water effectively prevent infection. In chronic wounds, such as diabetic foot ulcers and pressure injuries, antiseptic agents, including hypochlorous acid, sodium hypochlorite, polyhexanide, and citrate-based solutions, have shown promise in improving healing and reducing infection. In implant-based breast reconstruction and augmentation, data on antiseptics, such as chlorhexidine, and changes in FDA guidance for povidone–iodine and bacitracin have prompted a reevaluation of intraoperative irrigation practices. Conclusion: Despite widespread use, many antiseptics remain off-label, and high-quality clinical studies comparing efficacy and safety are lacking.

## 1. Introduction

Plastic and reconstructive surgeons routinely employ irrigation solutions across a range of clinical settings. In acute and chronic wound management, irrigation plays a vital role in mechanically and chemically reducing bioburden, disrupting biofilms, and removing necrotic tissue, creating a wound environment conducive to healing [1]. Irrigation is presumed important in implant-based breast surgery, where it aids in cleansing contaminated fields and preventing implant infections, a significant complication that increases patient morbidity and healthcare spending [2,3]. Saline is the most commonly used irrigation solution, and antiseptic and antibiotic solutions are used in situations where infection prevention and control are of concern. Growing emphasis on antibiotic stewardship has shifted practice away from antibiotic solutions and toward antiseptics, yet there are concerns about antiseptic cytotoxicity and antimicrobial resistance [4,5]. This article reviews irrigation solutions used in plastic surgery and examines their application and efficacy in both wound care and implant-based breast surgery, with particular focus on studies published in the last three years. It critically evaluates the efficacy, limitations, and clinical relevance of various solutions while identifying key knowledge gaps that warrant further investigation.

## 2. Methods

A broad literature review identified irrigation solutions, including antibiotics, saline, tap water, and antiseptics, commonly reported in plastic surgery and wound care. De-vices@FDA (https://www.accessdata.fda.gov/scripts/cdrh/devicesatfda/index.cfm), Drugs@FDA (www.fda.gov/drugsatfda), and DailyMed (https://dailymed.nlm.nih.gov/dailymed/) were searched to obtain information on labeled indications, FDA-cleared or -approved uses, and concentrations. PubMed (https://pubmed.ncbi.nlm.nih.gov), Cochrane Central Register of Controlled Trials (CENTRAL) via the Cochrane Library (https://www.cochranelibrary.com), and Elsevier’s Embase (https://www.embase.com) were searched to identify human clinical studies evaluating these irrigation solutions in acute wounds, chronic wounds, and implant-based breast surgery published between January 2022 and July 2025. All websites and databases were accessed between July–August 2025.

## 3. Irrigation Solutions

This section reviews the spectrum of irrigation solutions, including saline, potable tap water, antibiotics, and antiseptics. It focuses on antimicrobial efficacy, cytotoxicity profiles, and concerns for resistance. See Table 1 for a comprehensive summary.

### 3.1. Saline and Potable Tap Water

Sterile saline, typically prepared at a concentration of 0.9%, is the most widely used irrigation solution. It is isotonic relative to plasma and avoids osmotic shifts and hemolysis when applied to tissue [45]. Its effect stems from mechanical removal of debris with a pressurized stream and dilution of bioburden. The lack of antimicrobial properties minimizes the risk of microbial resistance and cytotoxicity [46].

Potable tap water provides similar mechanical cleansing and dilutional benefits despite being non-sterile. With appropriate chlorination and filtration, potable tap water can achieve sufficiently low bacterial counts to be considered safe for use in emergency settings [32,47]. However, due to its hypotonicity, tap water should generally be avoided for intracavitary lavage.

### 3.2. Antibiotics

Antibiotic irrigation has traditionally been used to reduce infection risk in surgical and wound care settings. Commonly used agents include bacitracin, vancomycin, gentamicin, cefazolin, polymyxin B, clindamycin, neomycin, and tobramycin [48,49,50,51].

Antibiotic irrigation may contribute to antimicrobial resistance, which the World Health Organization identifies as a leading global health threat: bacterial resistance was linked to an estimated 4.95 million deaths in 2019 [52,53]. Evidence supporting the efficacy of antibiotic irrigation remains limited, prompting increased scrutiny of its routine use and underscoring the need for judicious, evidence-based application [52,54]. Additionally, cytotoxicity remains a concern. For instance, an in vitro study showed that bacitracin, polymyxin B, and gentamicin had cytotoxic effects on human keratinocytes, suggesting that antibiotic irrigation may hinder wound healing [55].

### 3.3. Antiseptics

Antiseptic solutions have broad-spectrum antimicrobial properties. Compared to antibiotics, there is lower risk of antimicrobial resistance, but cytotoxicity remains a concern. This section reviews antiseptic solutions used in plastic surgery and wound care. Of note, silver nitrate was not included in this review. Although historically used in burn care, its role has diminished with the advent of modern silver-based dressings, which offer sustained antimicrobial activity with less tissue staining and electrolyte disturbances.

#### 3.3.1. Acetic Acid

Acetic acid is effective against *Pseudomonas aeruginosa* and other Gram-positive and Gram-negative bacteria. It is generally well tolerated at 0.25–5%, though higher concentrations may cause irritation [6]. Despite in vitro cytotoxicity concerns, clinical data show no impairment in wound healing [56]. While resistance has been noted in enteric bacteria in food preservation studies, evidence of resistance in wound care is limited [57].

#### 3.3.2. Chlorhexidine Gluconate and Polyhexanide

Chlorhexidine gluconate (CHG) and polyhexanide (PHMB) are biguanides with differing safety and efficacy profiles. Alcoholic CHG (2–4%), used for skin preparation, has been linked to delayed skin wound healing, while aqueous CHG (0.5–1%) is less cytotoxic [11,12,58,59]. Additionally, antimicrobial resistance and cross-resistance to antibiotics, such as ceftazidime, sulfamethoxazole, imipenem, cefotaxime, and tetracycline, are associated with CHG [60]. Emerging in vitro studies have also explored the adjunctive use of photodynamic therapy with CHG, suggesting potential synergistic antimicrobial effects [61]. Conversely, when directly compared in vitro, PHMB is more efficacious in reducing bioburden and biofilms with lower cytotoxicity [62]. In general, there is a low risk of antimicrobial resistance to PHMB, but it has been linked to gentamicin cross-resistance [63].

#### 3.3.3. Hydrogen Peroxide

Hydrogen peroxide (H_2_O_2_), once commonly used at 3% for wound cleansing, is now used sparingly due to its cytotoxicity and potential to delay healing. Endogenous H_2_O_2_ promotes inflammation, making topical use particularly undesirable in chronic wounds, which exhibit a prolonged inflammatory phase [64]. However, these adverse effects may be dose-dependent, warranting further investigation into optimal concentrations [65]. While some microbes have innate defenses against H_2_O_2_, there is limited evidence of true resistance development [66].

#### 3.3.4. Hypochlorous Acid, Sodium Hypochlorite, Super-Oxidized Solution, and Oxychlorosene

Hypochlorous acid (HOCl) and its conjugate base, sodium hypochlorite (NaOCl), are strong oxidants used for their antimicrobial and wound-healing properties. HOCl is used at 0.01–0.025% concentration and may be more efficacious and better tolerated than NaOCl, in part because human neutrophils make it during the respiratory burst, and human cells have protective mechanisms against it [21,24,67]. In contrast, NaOCl, used at 0.125–0.5%, can be caustic and may cause tissue irritation [42,68]. The effects of NaOCl on wound healing and its cytotoxicity are less clear; some in vitro studies report high cytotoxicity, while others simulating in vivo conditions observe minimal effects [69].

Direct comparative studies between HOCl and NaOCl are lacking. From a practical standpoint, NaOCl benefits from a longer shelf life, whereas HOCl degrades upon exposure to light and air, requiring careful storage. HOCl also quickly interacts with tissue proteins, potentially requiring higher concentrations to maintain efficacy [70]. Both HOCl and NaOCl have been associated with Gram-negative bacterial resistance and potential cross-resistance with antibiotics [60,71].

Super-oxidized solution contains 0.003% HOCl and 0.004% NaOCl and is prepared using electric current to increase ionic concentration. Oxychlorosene is a powder that slowly releases HOCl when dissolved in an aqueous solution, typically used at 0.2% concentration. These agents have minimal cytotoxicity and are associated with improved wound healing and reduced infection rates [25,72].

#### 3.3.5. Povidone–Iodine

Povidone–iodine (PVI), a water-soluble complex of iodine and the polymer povidone, remains widely used due to its broad-spectrum antimicrobial activity, including efficacy against biofilms and multi-drug-resistant bacteria [73]. Unlike other antiseptics, there is no documented microbial resistance or cross-resistance to PVI [74]. However, there are concerns regarding its tissue toxicity at high concentrations [75]. Consequently, efforts have focused on identifying an optimal concentration that preserves antimicrobial efficacy while minimizing tissue toxicity. In vivo studies in rat models determined that concentrations between 0.5 and 5% are effective in eliminating bacteria without causing thyroid, kidney or liver damage [75]. Other in vitro studies report no significant cytotoxic effects at concentrations below 10% while maintaining activity against key pathogens such as methicillin-sensitive *Staphylococcus aureus* (MSSA) [76].

#### 3.3.6. Citrate-Based Solution

Citrate-based solution is composed of 3.25% citric acid and 3.12% sodium citrate. In vitro, in vivo, and ex vivo studies support its antibiofilm properties, anti-inflammatory effects, and safety with bone, cartilage, and soft tissue. However, large randomized controlled trials (RCTs) to confirm its clinical efficacy are ongoing [10]. To date, there is limited evidence to suggest antimicrobial resistance associated with citrate.

## 4. Clinical and Surgical Challenges

### 4.1. Biofilms

Biofilms consist of microbial communities encased in an extracellular polymeric substance (EPS) that adheres to surfaces, such as chronic wounds and implants [77,78]. They are particularly difficult to treat because they evade host immune defenses, resist antimicrobial agents, and are difficult to identify upon visual inspection [78]. The presence of a foreign body, such as an implant, initiates a localized immune response that leads to fibrous encapsulation and creates a microenvironment with limited immune surveillance, while the EPS further impairs immune clearance and reduces antibiotic efficacy by limiting diffusion and neutralizing antimicrobial agents [78,79]. Systemic antibiotics are often insufficient to eradicate biofilms. Irrigation solutions are necessary to mechanically remove debris and planktonic (non-adhered) bacteria, while antiseptics also chemically disrupt biofilm structure and viability [77,80].

Antibiotic irrigation is generally avoided due to limited efficacy and high biofilm-associated resistance [81,82]. Common antiseptics include CHG, PHMB, PVI, HOCl, and NaOCl. In vitro studies show that CHG and PVI consistently reduce biofilms formed by *Staphylococcus epidermidis*, *Escherichia coli*, *Candida albicans*, *P. aeruginosa*, and MSSA [82,83,84,85]. PHMB has outperformed PVI both in vitro and in vivo, while also demonstrating lower cytotoxicity [86]. In contrast, HOCl and NaOCl show limited biofilm efficacy at low concentrations and without extended dwell time in vitro [86,87].

Detergent-enhanced formulations, such as PHMB with betaine, PHMB with poloxamer, or citrate with sodium lauryl sulfate, improve biofilm disruption and antimicrobial activity [10,88,89]. While antiseptics offer some efficacy, culture-directed antibiotic therapy is still recommended to narrow antimicrobial exposure and minimize toxicity [52].

### 4.2. Delivery Methods

Low- and high-pressure irrigation systems are methods for delivering wound irrigation, each with distinct clinical considerations. Low-pressure irrigation, typically delivered via gravity or a bulb syringe at ≤10 psi, is favored for its simplicity and minimal tissue trauma. In contrast, high-pressure pulsatile lavage (>10 psi), administered using a pulse irrigator or pulsatile lavage system, is employed for more effective mechanical removal of debris and reduction in bacterial burden [90,91]. However, in vitro and in vivo studies have raised concerns that high-pressure irrigation could drive bacteria deeper into tissues, potentially increasing infection risk and causing damage to surrounding structures [92,93]. As a result, current practice favors low- to moderate-pressure irrigation (5–15 psi), delivered via gravity or pressure-assisted devices, to balance effective debridement with tissue preservation [91,93,94].

Negative pressure wound therapy with instillation and dwell time (NPWTi-d) is an emerging method that combines wound irrigation with traditional negative pressure wound therapy (NPWT). In this system, a prescribed volume of an irrigation solution is intermittently delivered to the wound bed, allowed to dwell for a set period (typically 5–20 min), and then removed with negative pressure. It is used across a range of wound types for its ability to remove exudate, facilitate wound cleansing and debridement, and promote granulation tissue formation [95]. However, not all solutions are compatible with NPWTi-d, as some are cytotoxic or degrade foam dressings. Compatible agents include saline, HOCl, NaOCl, acetic acid, and PHMB [96]. Antiseptic solutions are typically reserved for wounds with high bioburden, where infection risk outweighs the potential for delayed healing due to cytotoxic effects [97].

### 4.3. Acute and Chronic Wounds

Acute wounds are injuries that progress through the normal stages of healing and typically result from surgery or trauma. Chronic wounds, such as diabetic foot ulcers (DFU), pressure injuries, and venous leg ulcers, are characterized by prolonged inflammation and impaired epithelialization. In both settings, irrigation is essential for debridement and reducing bacterial burden [1].

Early evidence supported the use of antibiotic irrigation in wound care. For instance, an RCT of 260 patients with lacerations demonstrated significantly lower infection rates with ampicillin irrigation compared to saline [98]. However, this approach never achieved widespread adoption. A systematic review later found that antibiotic solutions offered no clear benefit in chronic wound care and posed risks such as delayed healing, anaphylaxis, nephrotoxicity, and ototoxicity [99]. In 2017, the Centers for Disease Control (CDC) recommended against antibiotic wound irrigation due to limited efficacy and the risk of antimicrobial resistance [54]. Furthermore, no antibiotic solution is FDA-approved for wound irrigation [100]. As a result, antibiotic irrigation has largely been abandoned in favor of saline and antiseptics [101].

In both acute and chronic wound management, sterile saline remains a favored irrigation solution due to its non-cytotoxic profile and widespread availability [45]. However, potable tap water has emerged as a practical alternative, especially in emergency and low-resource settings, as sterile saline is approximately ten times more expensive [102]. Multiple RCTs conducted from the early 2000s through the 2010s consistently found no significant difference in wound infection rates between lacerations irrigated with sterile saline and those irrigated with potable tap water [102,103,104,105]. Reflecting this evidence, the American College of Emergency Physicians formally endorsed the use of potable tap water for acute wound irrigation in its 2024 guidelines [32].

PVI is FDA-cleared for use as a topical antiseptic at 5–10% concentration, but it is commonly used off-label in wound care at lower concentrations ranging from 0.35% to 5%. However, based on evidence, such as an RCT of 446 simple lacerations that found no significant difference in infection rates between irrigation with PVI 1% and saline, the American Heart Association and American Red Cross recommend against its use in acute wound care and instead endorse saline [106,107]. In contrast, PVI is valued in chronic wound care for its broad-spectrum antimicrobial activity and efficacy against biofilms. Additionally, some studies suggest PVI may have anti-inflammatory properties that promote healing. Nonetheless, concerns about cytotoxicity and potential systemic effects after prolonged use may limit its application in chronic settings [73].

The FDA has cleared several antiseptics for chronic wound management, including 0.05% aqueous CHG, 0.1% polyhexanide with 0.1% betaine, 0.025% HOCl, 0.125–0.5% NaOCl, super-oxidized solution, and citrate-based solution [13,18,24,31,42,43].

A systematic review and meta-analysis found that PHMB was associated with accelerated healing in chronic wounds compared to controls [108]. Several RCTs throughout the 2010s compared 0.1% PHMB with 0.1% betaine to saline. Two small RCTs studied its use in chronic lower extremity ulcers. One showed no significant change in bacterial burden or biofilm, while the other found no difference in wound size but noted significant reductions in wound pH (a proxy for bacterial colonization) and odor [109,110]. A larger RCT of 289 patients with pressure injuries or vascular ulcers demonstrated significantly faster healing with PHMB/betaine [111].

HOCl exhibits anti-inflammatory properties and has been associated with improved healing in chronic and infected wounds compared to saline [67]. A randomized pilot study of 16 patients with chronic wounds found that 0.057% NaOCl led to better healing than saline [112]. Similarly, an RCT of 100 DFUs showed that 0.1% NaOCl significantly reduced wound size and complications compared to saline [113]. Super-oxidized solution has also shown promise, particularly in DFUs, with studies demonstrating superior healing and infection reduction compared to PVI [72].

Lastly, acetic acid is often used in wounds infected with *P. aeruginosa*. An RCT of 32 chronic wounds demonstrated a significantly faster elimination of *P. aeruginosa* with 1% acetic acid irrigation compared to saline [114].

### 4.4. Implant-Based Breast Reconstruction and Augmentation

In implant-based breast reconstruction and augmentation, implant infection is a significant post-operative complication. Infection rates following primary breast augmentation range from 1% to 2.5%, whereas rates following implant-based breast reconstruction after mastectomy are higher, ranging from 5.4% to 35% [115,116,117]. Infection often requires hospitalization and reoperation, which increases morbidity, cost, and overall burden on the healthcare system [3,116,118]. In breast reconstruction patients, infections may delay adjuvant cancer therapy, potentially impacting oncologic outcomes [3]. Capsular contracture (CC), another clinically significant complication, has been linked to subclinical implant infections and biofilm formation [119].

To mitigate these risks, the breast pocket is irrigated intraoperatively. Historically, irrigation solutions have included PVI, antibiotics, and saline [120]. Then, in 2000, the FDA banned the use of PVI for breast pocket irrigation due to concerns for implant degradation [121]. As a result, triple antibiotic solution (TAS) of cefazolin, gentamicin, and bacitracin became the predominant irrigation solution [2,122,123]. In 2017, the FDA lifted its warning on PVI, acknowledging that implant shell integrity was not compromised by its use; however, this revision did not constitute a formal recommendation [2].

Due to these regulatory shifts, several studies have examined the efficacy of PVI breast pocket irrigation (see Table 2). A recent systematic review and meta-analysis of 27 studies supported PVI’s efficacy in reducing surgical site infections (SSIs) in implant-based breast augmentation [124]. A prospective study of 2088 patients (4176 breasts) found no significant difference in CC rates between TAS with and without PVI but recommended PVI for its broad Gram-negative coverage, given the association between Gram-negative colonization and breast implant-associated anaplastic large cell lymphoma (BIA-ALCL) [125]. PVI use remains off-label. It is essential that only sterile formulations are used. The FDA cautions that products not explicitly labeled as sterile, such as Betadine^®^ 10% stock solution, are non-sterile and intended solely for topical application [40,126]. We have observed a shift in institutional practice from the 10% stock solution to sterile formulations for intraoperative use.

CHG, HOCl-based, and citrate-based solutions are being explored as other antiseptic irrigation solutions. Two systematic reviews concluded that CHG is associated with low SSI and CC rates [124,140]. A small retrospective study of 53 patients (94 breasts) undergoing immediate post-mastectomy breast reconstruction (PMBR) with tissue expanders (TE) evaluated the efficacy of adding 0.05% CHG to a regimen of cefazolin, gentamicin, and PVI. The authors reported a significant reduction in bacteria on culture but no significant difference in SSIs. Additionally, a pivotal RCT that compared 0.05% CHG with TAS breast pocket irrigation for PMBR with TEs showed no significant difference in SSIs, though TAS approached significance for lower rates of infectious complications [16].

A retrospective review of 331 implant-based reconstructions comparing TAS to oxychlorosene, a derivative of HOCl, found no significant difference in infection rates. Oxychlorosene was favored for its lower cost and ease of use [25]. Additionally, citrate-based solutions have been effective in reducing SSIs in total joint arthroplasty and biofilms [10]. Currently, an RCT comparing Xperience^®^ to PVI in implant-based breast reconstruction is ongoing and could add to the evidence-based role for more modern antiseptic irrigation in implant-based breast surgery [19].

The landscape of breast pocket irrigation shifted in 2020 when the FDA recalled bacitracin injection due to risks of nephrotoxicity and anaphylaxis, effectively eliminating its use in TAS protocols [121]. In response, several studies evaluated outcomes in implant-based breast surgery before and after the recall. Two retrospective studies found no significant differences in SSI rates, while a larger NSQIP case–control study reported higher odds of SSI in the post-recall cohort [127,128,134]. The retrospective studies confirmed the use of bacitracin, but the NSQIP study was only able to use a temporal proxy for bacitracin use, raising concerns about misclassification. Tirrell et al. further detailed specific antibiotic and antiseptic combinations, including cefazolin, gentamicin, vancomycin, polymyxin B, and PVI. Notably, polymyxin B was used only after the recall, possibly suggesting a shift in surgeon preference to compensate for the absence of bacitracin [127].

While findings are mixed and the lack of standardized comparison groups makes drawing conclusions difficult, prospective trials are unlikely given the safety concerns surrounding bacitracin. Thus, future high-quality retrospective studies with detailed analyses of irrigation protocols are needed to more definitively assess the impact of bacitracin-based irrigation on surgical outcomes.

With the removal of bacitracin from clinical use and the improvement of surgical and sterile techniques since the widespread adoption of TAS in the early 2000s, it is timely to question the necessity of antibiotic irrigation. A retrospective study of 371 patients (445 breasts) undergoing immediate prepectoral PMBR compared a combination solution of PVI, gentamicin, and saline with and without cefazolin; no differences in SSI or CC were observed [133]. Two systematic reviews assessed antibiotic irrigation in primary breast augmentation: one found no significant difference in SSI or CC when compared to non-antibiotic irrigation, while the other reported a lower CC rate with antibiotic irrigation compared to saline or no irrigation [132,139]. A small RCT (16 patients, 32 breasts) conducted prior to the bacitracin recall comparing TAS to saline found no SSIs in either group, limiting conclusions [130].

Breast implant salvage is an emerging area of study with limited high-level evidence. A prospective pilot study reported a 100% salvage rate in 13 breasts using culture-guided antibiotic irrigation over one week [141]. Retrospective studies have also shown high salvage rates and low infection rates with 1–2 g vancomycin irrigation for 2–3 days [129,135]. Additionally, NPWTi-d with oxychlorosene has shown promise: a retrospective study of 81 patients (136 breasts) demonstrated significantly improved salvage rates and fewer implant-free days compared to controls [136]. While early findings are encouraging, larger studies are needed to validate these approaches and optimize protocols.

## 5. Limitations of Current Evidence

While in vitro studies are valuable for their cost-effectiveness and avoidance of ethical issues related to animal or human subjects, their findings should not be extrapolated to clinical contexts. For instance, results from in vitro and in vivo cytotoxicity studies of irrigation solutions often differ, likely due to the protective role of the extracellular matrix in in vivo models [86]. Dudek et al. found that PHMB had moderate cytotoxicity in vitro but then found no cytotoxicity in a larval model [86]. Additionally, HOCl quickly interacts with tissue proteins, reducing its active concentration. Thus, levels deemed harmful in vitro may be safe and necessary to reduce bioburden in clinical practice [70]. Establishing optimal concentrations is essential to achieve bacterial clearance while minimizing the risk of antimicrobial resistance [60,71].

In vivo models are also limited. For example, the larval model used in the Dudek et al. study is generally simpler than mammalian systems [86]. Therefore, clinical studies directly comparing irrigation solutions are crucial to fully understanding their cytotoxicity and impact on wound healing. Similarly, much of the evidence about irrigation solutions on biofilms comes from in vitro studies, and there are few high-quality clinical trials.

Although antiseptics like PVI are commonly used for breast pocket irrigation and wound care, they are often utilized for off-label indications. Despite its widespread use, few high-quality RCTs have directly compared PVI’s antimicrobial efficacy and cytotoxicity with other agents such as PHMB, which may offer comparable or superior performance with lower tissue toxicity. PHMB remains less studied in the U.S. compared to Europe, which has likely limited its widespread clinical adoption. There is a clear opportunity for industry to develop FDA-approved, standardized antiseptic irrigation solutions tailored for intraoperative use and wound care.

## 6. Conclusions

Irrigation remains a cornerstone of wound management and implant-based breast surgery, yet consensus on the optimal solutions remains elusive. In acute wounds, saline and potable tap water are both effective for reducing infections with minimal cytotoxicity and low cost. For chronic wounds, growing evidence supports the use of antiseptics, such as PHMB and HOCl-based solutions, which demonstrate promising antimicrobial and healing properties. Still, cytotoxicity remains a concern.

In breast pocket irrigation, the field continues to evolve following the FDA’s withdrawal of bacitracin. While PVI has regained popularity due to its broad-spectrum efficacy, concerns about tissue toxicity and the availability of potentially safer alternatives, such as citrate-based solutions, merit further exploration. Although antibiotic irrigation was widely used in the TAS era, its continued relevance is unclear. Conflicting evidence and a lack of standardized antibiotic formulations make it difficult to draw firm conclusions. Available data suggest that antiseptic-only regimens may offer comparable protection in many settings.

Across all indications, the key challenge is balancing antimicrobial efficacy with tissue preservation and minimizing resistance. Future research should prioritize comparative clinical studies evaluating different antiseptics at various concentrations. Establishing safe, effective irrigation strategies that are adaptable across clinical indications and resource settings is essential to advancing evidence-based care.

## Figures and Tables

**Table 1:** Irrigation solutions described in this review, listed alphabetically by generic name with corresponding brand names, typical concentrations, U.S. FDA-approved or -cleared indications, and other reported clinical uses.

Irrigation Solution	Example Brand Names	Concentration	FDA-Cleared/Approved Uses	Other Uses
Acetic acid		0.25%	Bladder irrigation with indwelling urethral catheter [6]	
	VoSoL^®^	2%	Superficial infections of the external auditory canal [7]	
		1–5%	None	Wound irrigation [8]
Antibiotics		Variable	None	Joint arthroplasty, peritoneal lavage, breast pocket [2,9,10]
Chlorhexidine gluconate	ChloraPrep^®^	2% (alcoholic)	Antiseptic skin preparation [11,12]	
	Hibiclens^®^	4% (alcoholic)		
	Desinclor^®^	0.05% (aqueous)	Cleansing and removal of debris, dirt, microorganisms from wounds [13]	Ophthalmic preparation, joint arthroplasty, breast pocket [14,15,16]
	Peridex^®^	0.12% (aqueous)	Oral rinse [17]	
Citrate-based solution	Xperience^®^	3.25% citric acid, 3.12% sodium citrate, 0.1% sodium lauryl sulfate	Cleansing and removal of debris and microorganisms from wounds [18]	Joint arthroplasty, breast pocket [10,19]
Hydrogen peroxide	Good Sense^®^	3%	None	First aid antiseptic, oral debriding agent [20]
Hypochlorous acid	PhaseOne^®^	0.01%	Skin abrasions, lacerations, minor irritations, cuts, and intact skin [21]	
		0.025%	None	Breast pocket, infected hardware salvage [22,23]
	Vashe^®^	0.025%	Acute and chronic wounds, first- and second-degree burns [24]	
Oxychlorosense	Clorpactin WCS-90^®^	0.2%	None	Breast pocket, bladder [25,26]
Polyhexanide	Serasept^®^	0.04%	None	Peritoneal lavage, surgical incisions, acute wounds [27,28,29]
		0.1–0.5%	None	Chronic wounds [30]
	Prontosan^®^	0.1% with 0.1% betaine	Chronic wounds, minor cuts, abrasions, lacerations, and minor burns [31]	
Potable tap water			None	Acute lacerations, wounds in low-resource settings [32,33]
Povidone–iodine		<1%	None	Chronic wounds, joint arthroplasty, peritoneal lavage [10,34]
		1%	None	Mucous membrane preparation, first aid antiseptic, surgical incisions [35,36]
	Betadine^®^	5%	Ophthalmic preparation *, antiseptic skin preparation [37,38,39]	Chronic wounds, peritoneal lavage, breast pocket [40]
		7.5–10%		Breast pocket [40]
Saline		0.9%	All general irrigation, washing, rinsing [41]	
Sodium hypochlorite	Dakin’s Wound Cleanser^®^	0.125%	Acute and chronic wounds, first- and second-degree burns, and grafted and donor sites, removal of dirt and debris [42]	
		0.25%		Breast pocket [2]
		0.5%		
Super-oxidized solution	Dermacyn^®^	0.003% HOCl, 0.004% NaOCl	Acute and chronic wounds, first- and second-degree burns [43]	Peritoneal lavage [44]

* 5% povidone–iodine only. Abbreviations: FDA, Food and Drug Administration; HOCl, hypochlorous acid; NaOCl, sodium hypochlorite.

**Table 2:** Human studies examining breast pocket irrigation solutions, categorized by experimental agent and listed in chronological order. Evaluated outcomes include SSIs, infectious complications, CC, and implant salvage rates.

Experimental (A)	Comparison (B)	Study Population	Study Type	Number of Subjects	Conclusion (A vs. B)	Year, Citation
Antibiotics						
Antibiotics excluding bacitracin, PVI	Antibiotics, including bacitracin, PVI	Implant-based breast reconstruction or augmentation	Retrospective	143 patients, 254 breasts	No significant difference in SSI	2022 [127]
After bacitracin recall	Before bacitracin recall	Implant-based PMBR	Case–control	12,626 patients	Increased odds of SSI	2023 [128]
Vancomycin for 2 days	Standard care: removal of implant, irrigation with saline, insertion of new implant	Immediate implant-based PMBR implant salvage	Retrospective	335 patients	All 7 patients in A had successful salvage and no complications	2023 [129]
TAS	Saline	Immediate PMBR with TEs	RCT	16 patients, 32 breasts	No SSIs reported	2023 [130]
Gentamicin or vancomycin	No irrigation	Implant-based breast reconstruction or augmentation	Retrospective	1508 patients	No significant difference in SSI	2024 [131]
Antibiotic irrigation	Non-antibiotic irrigation	Implant-based breast reconstruction or augmentation	Systematic review and meta-analysis	7 studies	No significant difference in SSI and CC	2024 [132]
Gentamicin, PVI	Cefazolin, gentamicin, PVI	Immediate PMBR with prepectoral implants	Retrospective	371 patients, 445 breasts	No significant difference in SSI or CC	2024 [133]
Non-bacitracin irrigation	Bacitracin irrigation	Implant-based PMBR	Retrospective	188 patients, 345 breasts	No significant difference in SSI	2024 [134]
Vancomycin for 2–3 days	NA	Implant-based PMBR implant salvage	Retrospective	37 patients	Improved implant salvage rate vs. literature values	2024 [135]
**Antiseptics**						
TAS	TAS, PVI	Implant-based breast augmentation	Prospective observational	2088 patients, 4176 breasts	No significant difference in CC	2023 [125]
NPWTi-d with oxychlorosene	No NPWTi-d	Implant-based PMBR implant salvage	Retrospective	81 patients, 136 breasts	Significantly increased implant salvage rate, significant reduction in days without implant	2024 [136]
CHG, cefazolin, gentamicin, PVI	Cefazolin, gentamicin, PVI	Immediate PMBR with TEs	Retrospective	53 patients, 94 breasts	Significant reduction in bacteria on culture, no significant difference in SSIs	2025 [137]
**Multiple solution types**						
TAS	CHG	Immediate PMBR with TEs	RCT	88 patients	No significant difference in SSIs	2021 [16]
No irrigation	NA	Implant-based breast augmentation	Retrospective	1128 patients	No difference in SSI rates compared to literature values for irrigation	2022 [138]
Antibiotics, PVI, CHG, HOCl	Saline, no irrigation	Implant-based breast augmentation	Systematic review and meta-analysis	14 studies	Significantly lower CC rates with antibiotics vs. saline and no irrigation, and with PVI vs. saline	2022 [139]
Saline, PVI, CHG, TAS	NA	Implant-based breast augmentation	Systematic review and meta-analysis	27 studies	PVI was the most effective in reducing infection frequency, TAS was the most effective in reducing CC	2025 [124]

Abbreviations: PVI, povidone–iodine; SSI, surgical site infection; PMBR, post-mastectomy breast reconstruction; TE, tissue expander; RCT, randomized controlled trial; TAS, triple antibiotic solution; CC, capsular contracture; NA, not applicable; NPWTi-d, negative pressure wound therapy instillation and dwell time; CHG, chlorhexidine gluconate.

## Data Availability

No new data were created or analyzed in this study. Data sharing is not applicable to this article.

## Abbreviations

The following abbreviations are used in this manuscript:

FDA	United States Food and Drug Administration
HOCl	Hypochlorous acid
NaOCl	Sodium hypochlorite
PVI	Povidone–iodine
CHG	Chlorhexidine gluconate
PHMB	Polyhexamethylene biguanide (also called polyhexanide)
H_2_O_2_	Hydrogen peroxide
MSSA	Methicillin-sensitive *Staphylococcus aureus*
RCT	Randomized controlled trial
EPS	Extracellular polymeric substance
NPWTi-d	Negative pressure wound therapy with instillation and dwell time
NPWT	Negative pressure wound therapy
CDC	Center for Disease Control and Prevention
DFU	Diabetic foot ulcer
CC	Capsular contracture
TAS	Triple antibiotic solution
SSI	Surgical site infection
PMBR	Post-mastectomy breast reconstruction
TE	Tissue expander
BIA-ALCL	Breast implant-associated anaplastic large cell lymphoma
